# Nutrition Literacy Mediates the Relationships between Multi-Level Factors and College Students’ Healthy Eating Behavior: Evidence from a Cross-Sectional Study

**DOI:** 10.3390/nu13103451

**Published:** 2021-09-29

**Authors:** I-Ju Lai, Li-Chun Chang, Chia-Kuei Lee, Li-Ling Liao

**Affiliations:** 1 Department of Nutrition, I-Shou University, Kaohsiung City 824, Taiwan; laii@isu.edu.tw; 2 School of Nursing, Chang Gung University of Science and Technology, Taoyuan City 333, Taiwan; lichunc61@yahoo.com.tw; 3 Department of Ophthalmology, Chang Gung Memorial Hospital, Keelung City 204, Taiwan; 4 Department of Nursing, College of Medicine, National Cheng Kung University, Tainan City 701, Taiwan; chiakuei@mail.ncku.edu.tw; 5 Department of Health Management, I-Shou University, Kaohsiung City 824, Taiwan

**Keywords:** nutrition literacy, social ecological model, college students, healthy eating behavior

## Abstract

College students experience new pressures and choices as they transition to independent living and can easily develop unhealthy eating habits, resulting in obesity and obesity-related chronic diseases in later life. This study aimed to test the hypothesis that nutrition literacy (NL) mediated the relationship between multi-level factors influencing healthy eating behavior identified from the social-ecological model and healthy eating behavior of college students. A four-part questionnaire was completed by 412 participants recruited from six different four-year universities in Taiwan (effective response rate = 85.8%). Data were analyzed using descriptive statistics, an independent samples t-test, hierarchical multiple regression, and mediation analysis. The results indicated that the students’ mean nutrition literacy score was 4.32 (SD = 0.78, range = 1–6). In the social-ecological framework, nutrition literacy significantly predicted healthy eating behavior (β = 0.28, *p* < 0.001; Δ*F* = 32.54, *p* < 0.001; Δ*R*^2^ = 0.05) with control variables of background, intrapersonal, interpersonal, environmental, and macrosystem factors. Nutrition literacy mediated the effects of seven factors on healthy eating behavior across four levels. These findings suggested that strengthening influential multi-level factors associated with healthy eating behavior not only enhanced NL, but also improved individuals’ healthy eating behavior.

## 1. Introduction

Chronic diseases are a major cause of death in modern society, and some types of chronic diseases are related to overweight and obesity caused by unhealthy dietary patterns [1,2]. College students are in a period of transition to independent living [3,4], whereby they experience new environments and face new types of pressures [5]. Therefore, they may easily develop unhealthy eating habits and are more likely to develop obesity and obesity-related diseases in adulthood, which increases the risk of chronic diseases [4,6,7,8,9].

The social-ecological framework was based on the theory of ecosystems (i.e., ecological model) proposed by Bronfenbrenner [10,11] for the study of human development, which emphasized the interaction between humans and their environments. This theory posits that the entire ecological system in which growth occurs should be considered when understanding the process of human development. This perspective was subsequently applied to the exploration of health behavior, and researchers have suggested that changes in individuals’ health behavior were influenced by not only intrapersonal factors but also the interaction between individuals and their living contexts, including social, cultural, economic, and environmental factors. Therefore, when examining factors influencing health behavior, various features should be considered at different levels to provide a comprehensive explanation framework [12,13]. Regarding changing individuals’ dietary patterns or obesity problems, the theory of social ecology has been used by many scholars to explain, predict, and change the relationships between individuals’ eating behavior and sociocultural, policy-related, and physical environments [14,15,16,17,18,19]. Several qualitative studies have examined factors influencing the dietary behaviors of college students via interviews, including multi-level barriers and enablers. The results revealed that the factors influencing healthy eating in college students included individual factors (e.g., taste preferences, lack of discipline, time, improved food knowledge and education, meal planning, and involvement in food preparation), social networks (e.g., social support from parents and peers), physical environments (e.g., product prices, limited budgets, and the availability and accessibility of healthy and unhealthy foods), and macrosystems [20,21,22,23,24] (e.g., media and advertising).

From the social ecology perspective, college students’ unhealthy eating behavior is related to unfavorable factors at all levels. Therefore, it is necessary to cultivate students’ ability to adapt to the daily dilemma of dietary choices. In addition, the enhancement of nutrition literacy (NL) in undergraduates is a promising solution to the problem at hand [25,26,27,28]. NL, a type of health literacy [29], refers to the ability to obtain, process, and understand the nutritional information and skills required to make appropriate nutrition-related decisions [30,31,32]. Several studies have indicated that health literacy mediated the relationship between sociodemographic variables and health-promoting behaviors/health outcomes [33,34,35]. Since NL is associated with healthy eating behavior and derived from health literacy [28,36,37,38], and sociodemographic variables are also predictors of NL [38,39], we assume that NL may play a similar role to health literacy in mediating the effect of factors influencing healthy eating on healthy eating behavior.

To our knowledge, the pathways of association between multi-level factors related to healthy eating, NL, and healthy eating behavior in college students have not yet been examined. Therefore, in the present study, it was hypothesized that NL was a mediator of the relationship between multi-level factors influencing healthy eating behavior identified from the social-ecological model and healthy eating behavior of college students.

## 2. Materials and Methods

### 2.1. Sample Selection

A convenience sample consisting of 480 college students was recruited from 6 4-year universities in northern, central, and southern Taiwan (i.e., one national and one private university in each region). To be eligible for this study, participants had to be currently enrolled college students and taking at least 1 course.

### 2.2. Recruitment and Procedures

A total of 80 undergraduate students were recruited from each school, with a maximum of 45 students in each academic discipline. In addition, the total number of students with medical-, public health-, and nutrition-related majors in each school were limited to 45, to prevent their majors from influencing the study results.

Trained research assistants approached students on campus, provided information regarding the study, and invited them to participate. Upon providing written informed consent, students were asked to complete a self-administered questionnaire that measured NL, factors influencing healthy eating behavior, healthy eating behavior, and demographic information, and took approximately 20 min to complete. Participants were informed that they could withdraw from the study at any stage without incurring adverse personal consequences. In addition, they received a small gift as compensation for participation. All study procedures were approved by the Human Research Ethics Committee. In total, 480 questionnaires were returned, and 412 were valid (valid response rate: 85.8%). Questionnaires that met the following criteria were considered completed: the total number of missing data did not exceed one-third of the length of the questionnaire, and the questionnaire passed the deception detection test.

### 2.3. Instruments

The instrument was divided into 4 parts: healthy eating behavior scale, NL scale, factors influencing healthy eating behavior scale, and demographic characteristics scale. The first draft of the questionnaire was reviewed by 6 experts in nutrition-related fields for the significance of each item and their relevance to the measurement. When an expert was invited to review, they would receive a description of the research plan and the scoring scheme for the review. A 4-point scale was adopted: 1 point meant “inappropriate,” 2 points meant “significant modification needed,” 3 points meant “minor modification needed,” and 4 points meant “appropriate.” The content validity index (CVI) was calculated based on the assessment results of the experts [40]. After the review was concluded, 207 college students were recruited to pretest the instrument. Cronbach’s α was calculated to demonstrate internal reliability, and intraclass correlation coefficient (ICC) was calculated to reveal the test-retest reliability for a two-week interval.

Healthy eating behavior was assessed using a 13-item scale that measured the frequency with which the following types of healthy eating behaviors were practiced: a balanced diet (8 items), processed food consumption (1 item), nutrition label use (3 items), and healthy food choices (1 item). Responses were rated on a scale ranging from 1 (never) to 5 (always). Item scores were summed to provide a total score, and higher scores indicated higher levels of healthy eating behavior. This scale showed good reliability and content validity; Cronbach’s α was 0.86, ICC was 0.49 (*p* < 0.001), and the average content validity index (CVI) was 0.99.

NL was measured using an 8-item scale that measured capacity for processing nutritional information in the following five dimensions, based on the definition of NL [30,31,41]: obtain (i.e., the capacity to search for, locate, and acquire nutritional information; 2 items), understand (i.e., basic nutritional knowledge and the capacity to understand general nutritional information; 2 items), analyze (i.e., the capacity to discriminate between and analyze different types of nutritional information in a given situation; 1 item), appraise (i.e., the capacity to judge and assess nutritional information according to personal need; 2 items), and apply (i.e., the capacity to apply nutritional information to daily life, to maintain a healthy diet; 1 item). Responses were provided using a Likert scale ranging from 1 (very difficult) to 6 (very easy). Item scores were summed to provide a total score, and higher scores indicated higher levels of NL. This scale has undergone a rigorous development process [42], and the complete version of the scale is presented in the Supplementary Materials. It showed good reliability and content validity: Cronbach’s α was 0.93, ICC was 0.53 (*p* < 0.001), and the average CVI was 1.00.

Factors influencing healthy eating behavior were measured using a 34-item scale that measured the status of 13 factors on the following 4 levels of influences, based on the social-ecological framework: individual (intrapersonal), social (interpersonal), environmental (physical environmental or community settings), and macrosystem [43]. Furthermore, we included questions to identify deception to ensure high-quality questionnaire data. That is, the item “I prefer sugar-sweetened beverages to water” was modified as “I prefer water to sugar-sweetened beverages,” and both items were included in the questionnaire. Questionnaires returned with consistent responses for these 2 items were included in further analyses.

The items measuring factors influencing healthy eating behavior, such as preference for healthy food, healthy eating attitude and self-efficacy, emotional eating, mindless eating, social eating, and the availability of healthy and unhealthy food, were taken from the Multidimensional Home Environment Scale (MHES) [44]. Furthermore, factors influencing eating behavior in college students, as described in the literature, were also adopted [21,22,23,24]. The item selection and measurement of the scale were compiled by the research team, which included nutrition and health professionals, to meet the 4-level concept of the social-ecological model and echo the actual diet situation of Taiwanese college students. The items were adopted only when the experts reached a consensus, and the college students participating in the pre-test confirmed that none of the items had cultural adaption issues.

Responses were provided using a Likert scale ranging from 1 (strongly disagree) to 6 (strongly agree). Item scores were summed to provide a total score for each factor, and higher scores indicated stronger tendencies toward each factor. Table 1 shows the scale items and reliability and validity of the scale in measuring factors influencing healthy eating behavior. This scale showed good reliability and content validity: Cronbach’s α, ICC, and CVI for each factor are shown in Table 1. Moreover, Table 2 shows that the square root of average variance extracted (AVE) of each level in this scale was greater than its correlations with any other level in the model assessed, which indicates that the discriminant validity in this scale was acceptable [45].

Demographic characteristics, including sex, years at college, residence (on or off campus during the semester), and daily dietary expenses (average daily cost of food), were measured to control for alternative explanations regarding the relationships between NL and factors influencing healthy eating behavior.

### 2.4. Statistical Analysis

Statistical analyses were performed using SPSS 18.0. Descriptive statistics were calculated to provide information regarding participants’ characteristics. Hierarchical multiple regression, which included 6 steps (control variables, intrapersonal variables, interpersonal variables, environmental variables, macrosystem variables, and NL), was performed to estimate the prediction of healthy eating behavior. Mediation analysis was conducted to determine whether NL mediated the effects of factors influencing healthy eating behavior on healthy eating behavior. We adopted Baron and Kenny’s [46] procedure to assess the mediating variable. The variables were regarded as mediators if the following 3 criteria were fulfilled: (1) the independent variable affected the dependent variable (M1), indicating that the total effect was significant; (2) the independent variable affected the mediating variable (M2); and (3) the independent and mediating variables both affected the dependent variable, and the effect of the mediating variable was significant (M3). If 3 regression models were firmly established, the mediation effect existed. Subsequently, the Bootstrap method was used to verify the significance of this indirect effect. Through 5000 repeated samples, a bias-corrected (BC) confidence interval was constructed for the indirect effect. If the 95% confidence interval does not exceed 0, it signifies an indirect effect (or that the hypothesis of the intermediary effect has been verified) [47], and that the ratio of indirect to total effects has been calculated. The mediating effects of NL on the influence of various independent variables on healthy eating behavior were examined in accordance with the above procedure. All analyses included demographic characteristics (i.e., sex, years at college, residence, and daily dietary expenses) as covariates.

## 3. Results

### 3.1. Participant Characteristics

In total, 412 college students were included in the study (218 women, 53.6%; 189 men, 46.4%). The participants’ mean age was 20.12 (SD = 1.8) years. Almost 33% of participants were aged 19 years, and 22.3% were aged 20 years. Regarding living conditions during the semester, most students (66.3%) lived in dormitories (without kitchens), while 20.0% lived with their families, and 13.8% rented houses outside the school. Regarding daily dietary expenses, 206 and 145 students spent NTD$ 100–199 and 200–299 on food per day, respectively.

### 3.2. Nutrition Literacy and Healthy Eating Behavior

The mean score for NL was 4.32 (SD = 0.78), which was between “somewhat easy” and “easy” on the six-point scale. The mean score for healthy eating behavior was 3.13 (SD = 0.57), which was close to the midpoint on the five-point scale. This indicated that the mean frequency with which students practiced healthy eating behavior was close to “sometimes” (i.e., 3–4 days per week).

### 3.3. Predictive Effect of Nutrition Literacy on Healthy Eating Behavior

As shown in Model 6 in Table 3, NL predicted healthy eating behavior with all the influencing factors controlled for (β = 0.28, *p* < 0.001; Δ*F* = 32.54, *p* < 0.001; Δ*R*^2^ = 0.05). This result indicated that with background, intrapersonal, interpersonal, environmental, and macrosystem factors controlled for, NL still significantly predicted healthy eating behavior.

### 3.4. The Mediating Effect of Nutrition Literacy

**Figure 1:**
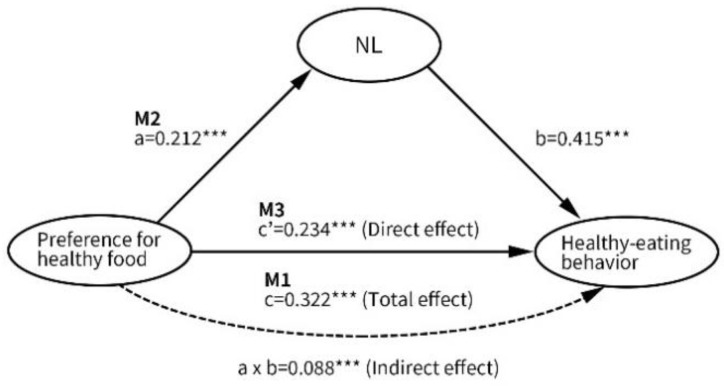
A graphical example of the mediating effect of NL, (*** *p* < 0.001).

Table 4 illustrates the results regarding the mediating effect of NL on the influences of various independent variables on healthy eating behavior. The analysis of total effects indicated that only four (emotional eating, social eating, availability of unhealthy food, and exposure to food-related advertising) of 13 influential factors were not significantly associated with healthy eating behavior. With regard to the analysis of mediating effects, the factors that were mediated by NL on an intrapersonal level included a preference for healthy food, healthy eating attitude, and healthy eating self-efficacy. The proportions of the total effects of preference for healthy food, healthy eating attitude, and healthy eating self-efficacy explained by mediation were 27.3%, 41.4%, and 25.9%, respectively. A graphical example of the mediating effect of NL is presented in Figure 1. On an interpersonal level, the factors that were mediated by NL included peer social support and family social support. The proportions of the total effects of peer social support and family social support explained by mediation were 31.0% and 48.3%, respectively. On an environmental level, only the availability of healthy foods was mediated by NL, and the proportion of the total effect of the availability of healthy foods explained by mediation was 43.1%. On a macrosystem level, only exposure to healthy eating campaigns was mediated by NL, and the proportion of the total effect of exposure to healthy eating campaigns explained by mediation was 42.4%.

## 4. Discussion

Existing literature indicates that NL is significantly related to sociodemographic variables and healthy eating behavior [28,36,37,38,39]. Many scholars have used the social-ecological model to identify multi-level factors that affect healthy eating behavior [14,15,16,17,18,19]. To our knowledge, this is the first study to explore the mediating effect of NL on the relationship between healthy eating behavior and its influencing factors, incorporating the social-ecological model. The present study provides further evidence that NL is not only an independent predictor of healthy eating behavior but also a mediator of the effect of multi-level factors influencing the healthy eating behavior identified from the social-ecological model on healthy eating behaviors of college students.

Our hypothesis that NL was a mediator of the relationship between multi-level factors influencing healthy eating behavior identified from the social-ecological model and healthy eating behavior of college students was partly supported. According to the definition of the mediating effect in this study, these constructs should both satisfy the criterion of M1, M2, and M3 were all statistically significant in the hierarchical regression analysis, and in the Sobel test, the indirect effects of mediation variables should also be statistically significant. Seven out of the 13 factors influencing college students’ healthy eating behavior in four levels of the social-ecological model met the conditions mentioned above for determining the existence of the mediating effect. Thus, NL mediated the effects of seven factors on healthy eating behavior, and the ratio of indirect to total effects was calculated in the Sobel test to indicate the proportions of the total effects explained by the mediating effect of NL. The proportions of the seven factors mediated by NL were listed in descending order: 48.3% for family social support, 43.1% for availability of healthy foods, 42.4% for exposure to healthy eating campaigns, 41.4% for healthy eating attitude, 31.0% for peer social support, 27.3% for preference for healthy food, and 25.9% for healthy eating self-efficacy.

A further review of the factors that were most strongly mediated by NL at each level is provided below. On an interpersonal level, the proportion of the total effect of family social support explained by NL was the highest at 48.3%. Although only 20% of participants lived with their families during university study, the results implied that if students’ families valued the importance of a healthy diet during their childhoods, it could lead to the development of good NL [48,49]. Moreover, even if students left their families and lived independently, these values could exert a positive effect on their healthy eating behavior. On an environmental level, the proportion of the total effect of the availability of healthy foods explained by NL was the highest at 43.1%, while on a macrosystem level, the proportion of the total effect of exposure to healthy eating campaigns is explained by NL was the highest at 42.4%. These results indicated that a university campus with a good eating environment and adequate healthy eating campaigns could enhance the NL of students and thus effectively improve healthy eating behavior in college students. From this perspective, health-promoting strategies in schools [50], that actively improve environmental support regarding college students’ eating behavior via the setting approach, and have been promoted in Taiwan in recent years constitute an appropriate direction for interventions to enhance college students’ NL. On an intrapersonal level, the proportion of the total effect of healthy eating attitudes explained by NL was the highest at 41.4%. The impact of personal attitudes on behavior was explored in the traditional knowledge-attitude-practice model in previous research [51,52]. However, the impact of healthy eating attitudes on behavior occurred largely via the enhancement of NL. That is, college students’ NL should be strengthened by their positive attitudes toward a healthy diet, such as those involving enhancement of their belief in a healthy diet or the use of value clarification. Healthy eating behavior could ultimately be enhanced via this process.

Contrarily, two factors on the intrapersonal level (i.e., preference for healthy food and healthy eating self-efficacy) were less affected by the mediation of NL, suggesting that these two factors had a relatively stronger power to directly affect healthy eating behavior than the other five factors in the present study, or that there were other influences from unknown factors that needed to be further studied. Food preferences are malleable. If the goal is to improve people’s acceptance of healthier foods, then promoting the development of healthy food preferences early in life may be an important direction for future research [53]. Additionally, healthy eating self-efficacy gives people the necessary confidence in their ability to engage in healthy eating behavior, and it is gained through knowledge, understanding, and skill development [54]. Previous studies suggested a complex role of self-efficacy in affecting healthy eating behavior [55,56]. More research is needed in the future to confirm whether there are factors that affect the relationship between healthy eating self-efficacy and healthy eating behavior.

The findings of this study suggested that strengthening influential multi-level factors associated with healthy eating behavior not only enhanced NL, but also improved individuals’ healthy eating behavior. This study found that college students’ healthy eating behavior was not ideal, which was also reported in previous studies [3,4,57]. In the future, the use of the social-ecological model to develop nutrition intervention, whether to directly improve healthy eating behavior or indirectly improve healthy eating behavior through enhancing NL, is a feasible way to improve the health status of college students. Evidence provided by this research can help to prioritize contributing factors when developing interventions with a focus on improving healthy eating behavior.

The study was subject to the following limitations: convenience sampling and discrepancies in self-reported data. The results are not generalizable to all college students in Taiwan because we used convenience sampling, and the sample size was rather small. In addition, because this study used self-reported data, there may have been discrepancies in the participants’ subjective perceptions and actual practice, which may have led to errors in the interpretation of the results. Future studies can use objective measures to evaluate NL or subjective assessments supplemented by NL objective testing to avoid this shortcoming. Despite these limitations, this study adopted a rigorous research design, including valid and reliable measures, more than 400 participants from six universities in Taiwan, and strict statistical procedures, to reduce possible research biases.

## 5. Conclusions

This was the first study to examine the mediating effect of NL on the relationship between college students’ healthy eating behavior and related influential factors. This finding could be used as the empirical basis of the application of NL to the design of nutrition-based educational interventions. Future studies trying to adopt the social-ecological model to develop nutrition intervention to promote healthy eating behavior in college students should focus on constructs that may enhance students NL, such as family social support, availability of healthy foods, exposure to healthy eating campaigns, healthy eating attitude, and peer social support. While strengthening constructs, such as preference for healthy food and healthy eating self-efficacy, may directly affect healthy eating behavior, it will not be as heavily affected by NL.

## Figures and Tables

**Table 1:** Reliability and validity of the scale measuring factors influencing healthy eating behavior.

Factors	Example	Item	CVI	Cronbach’s α	ICC
**Intrapersonal Level**					
Preference for healthy food ^a^	I prefer fruit juice to fresh fruit	7	1	0.742	0.850 **
Healthy eating attitude	I think that it’s nice to have a healthy diet	2	1	0.865	0.692 **
Healthy eating self-efficacy	I’m sure that I can maintain a healthy diet	2	1	0.882	0.634 **
Emotional eating	When I am sad, I eat and vent my emotions	4	1	0.892	0.762 **
Mindless eating	I often do other things while eating	1	1	-	0.667 **
Healthy eating difficulty	I think it takes time to maintain a healthy diet	3	1	0.759	0.584 **
**Interpersonal Level**					
Social eating	I often dine with others	2	1	0.782	0.723 **
Peer social support	My friends encourage me to adopt a healthy diet	2	1	0.862	0.394 **
Family social support	My family members encourage me to adopt a healthy diet	2	1	0.944	0.703 **
**Environmental Level**					
Availability of healthy foods	It is easy to buy vegetables where I live or at school	3	1	0.782	0.695 **
Availability of unhealthy foods	It is easy to buy fast food where I live or at school	3	1	0.882	0.465 **
**Macrosystem Level**					
Exposure to food-related advertising	I often watch food-related advertisements or shows	2	1	0.832	0.651 **
Exposure to healthy eating campaigns	I am often exposed to messages regarding a healthy diet	1	1	-	0.434 **

Note: ^a^ Seven items within this factor were reverse scored. CVI = content validity index; ICC = intraclass correlation coefficient. ** *p* < 0.01.

**Table 2:** Discriminant validity of the scale measuring factors influencing healthy eating behavior.

	1	2	3	4
1. Intrapersonal Level	**0.517**			
2. Interpersonal Level	0.444 *	**0.599**		
3. Environmental Level	0.226	0.379 *	**0.729**	
4. Macrosystem Level	0.353	0.420	0.207	**0.674**

Note: The bold numbers on the diagonal are the square root of AVE, the other numbers are the correlations. * *p* < 0.05.

**Table 3:** Predictive effect of nutrition literacy on healthy eating behavior.

	Model 1	Model 2	Model 3	Model 4	Model 5	Model 6
Δ*R*^2^	0.039	0.278	0.004	0.012	0.025	0.054
Δ*F*	2.129 *	24.864 **	0.701	3.148 *	7.080 **	32.542 ***
Δ*p*	0.040	0.000	0.552	0.044	0.001	0.000
*R*^2^ (Final)	0.039	0.317	0.321	0.333	0.358	0.411
*F* (Final)	2.129 *	13.063 **	10.719 ***	9.991 ***	10.003 ***	11.913 ***
*p* (Final)	0.040	0.000	0.000	0.000	0.025	0.054

Note: Predictors were added in the following order: Model 1: control variables; Model 2: intrapersonal variables; Model 3: interpersonal variables; Model 4: environmental variables; Model 5: macrosystem variables; Model 6: nutrition literacy. *** *p* < 0.001, ** *p* < 0.01, * *p* < 0.05.

**Table 4:** Summary of the Mediating Effects of Nutrition Literacy.

Variables	Mediator	Dependent		BC 95% CI	Mediation Effect %
	Nutrition Literacy	Healthy Eating Behavior			
	M2	M1	M3		
Effect		Total Effect	Direct Effect	Indirect Effect	Indirect Effect/Total Effect
**Intrapersonal Level**					
IV: Preference for healthy food	0.212 ***	0.322 ***	0.234 ***	0.088 †	27.3%
Mediator: Nutrition literacy			0.415 ***	(0.038~0.135)	
Δ*R*^2^ (Δ*F*)			0.158 (84.21 ***)		
IV: Healthy eating attitude	0.403 ***	0.362 ***	0.212 ***	0.150 †	41.4%
Mediator: Nutrition literacy			0.374 ***	(0.101~0.205)	
Δ*R*^2^ (Δ*F*)			0.112 (58.21 ***)		
IV: Healthy eating self-efficacy	0.381 ***	0.478 ***	0.354 ***	0.124 †	25.9%
Mediator: Nutrition literacy			0.327 ***	(0.082~0.177)	
Δ*R*^2^ (Δ*F*)			0.087 (58.38 ***)		
IV: Emotional eating	0.103 *	0.093	0.046	0.047	-
Mediator: Nutrition literacy			0.459 ***	(−0.019~0.092)	
Δ*R*^2^ (Δ*F*)			0.200 (99.64 ***)		
IV: Mindless eating	0.026	−0.102 *	−0.114 *	0.012	-
Mediator: Nutrition literacy			0.466 ***	(−0.055~0.049)	
Δ*R*^2^ (Δ*F*)			0.208 (105.58 ***)		
IV: Healthy eating difficulty	−0.056	−0.102 *	−0.076	−0.026	-
Mediator: Nutrition literacy			0.459 ***	(−0.096~0.021)	
Δ*R*^2^ (Δ*F*)			0.201 (101.03 ***)		
**Interpersonal Level**					
IV: Social eating	0.114 *	0.089	0.037	0.052	-
Mediator: Nutrition literacy			0.459 ***	(−0.019~0.090)	
Δ*R*^2^ (Δ*F*)			0.200 (99.58 ***)		
IV: Peer social support	0.214 ***	0.290 ***	0.200 ***	0.090 †	31.0%
Mediator: Nutrition literacy			0.419 ***	(0.053~0.154)	
Δ*R*^2^ (Δ*F*)			0.161 (84.24 ***)		
IV: Family social support	0.220 ***	0.201 ***	0.104 *	0.097 †	48.3%
Mediator: Nutrition literacy			0.441 ***	(0.044~0.147)	
Δ*R*^2^ (Δ*F*)			0.177 (89.52 ***)		
**Environmental Level**					
IV: Availability of healthy food	0.161 **	0.167 **	0.095 *	0.072 †	43.1%
Mediator: Nutrition literacy			0.449 ***	(0.028~0.132)	
Δ*R*^2^ (Δ*F*)			0.189 (94.99 ***)		
IV: Availability of unhealthy food	0.168 **	−0.010	−0.089	0.079 †	-
Mediator: Nutrition literacy			0.476 ***	(0.005~0.109)	
Δ*R*^2^ (Δ*F*)			0.213 (105.54 ***)		
**Macrosystem Level**					
IV: Exposure to food-related advertising	0.083	0.056	0.020	0.038	-
Mediator: Nutrition literacy			0.460 ***	(−0.030~0.084)	
Δ*R*^2^ (Δ*F*)			0.203 (99.68 ***)		
IV: Exposure to healthy eating campaigns	0.327 ***	0.309 ***	0.178 ***	0.131 †	42.4%
Mediator: Nutrition literacy			0.403 ***	(0.081~0.192)	
Δ*R*^2^ (Δ*F*)			0.140 (71.43 ***)		

Note: sex, age, residence, and daily dietary expenses were controlled for. Unmarked numbers are standardized regression coefficients (β). M1: The relationship between the independent and dependent variables; M2: The relationship between the independent and mediating variables; M3: The combined effect of the independent and mediating variables on the dependent variable. IV = independent variable, BC = bias-corrected; * *p* < 0.05, ** *p* < 0.01, *** *p* < 0.001; † Significant by 5000 Bootstrap samples, biased corrected methods: 95% CI of standardized coefficient.

## Data Availability

The data presented in this study are available on request from the corresponding author. The data are not publicly available due to privacy and ethical concerns, neither the data nor the source of the data can be made available.

## Supplementary Materials

The following are available online at https://www.mdpi.com/article/10.3390/nu13103451/s1, Table S1: Items of the Nutrition Literacy (NL) Scale.
