# Supplementary material for: Nutrition Literacy Mediates the Relationships between Multi-Level Factors and College Students’ Healthy Eating Behavior: Evidence from a Cross-Sectional Study
Source: Nutrients. 2021 Sep 29;13(10):3451. doi: 10.3390/nu13103451 (PMC8539523; doi:10.3390/nu13103451)
Supplement: Supplementary file 1 [file nutrients-13-03451-s001.zip › nutrients-1361917-supplementary.pdf]

Supplementary Materials:

Table S1: Items of the Nutrition Literacy (NL) Scale

Items
Obtain
1. For me, when there are nutrition-related issues, knowing where to find the right information is …
2. For me, when I want to learn healthy-eating behaviors, knowing where to find the right information is …
Understand
3. For me, being able to understand the contents of the Daily Food Guide is …
4. For me, being able to understand the contents of the Dietary Guidelines for Taiwanese is …
Analyze
5. For me, choosing foods from the nutritional point of view to distinguish food groups and functions is …
Appraise
6. For me, judging whether the nutrition information on the network is correct or not is …
7. For me, choosing a method that meets my health need when there are many recommendations for healthy diets is …
Apply
8. For me, using the right nutrition information in daily life for healthy eating is …

Note. A 6-point Likert scale ranging from 1 (very difficult) to 6 (very easy) was employed and higher scores reflected better NL.
